# Editorial: Integrative perspectives on the student-athlete experience: a multi-disciplinary focus

**DOI:** 10.3389/fspor.2026.1835996

**Published:** 2026-04-08

**Authors:** Eddie G. Walker II, Anas Al-Fattal

**Affiliations:** Business Department, University of Minnesota Crookston, Crookston, MN, United States

**Keywords:** cross-cultural, dual-career athletes, mental health, post-athletic career, student-athletes

Some athletes around the world participate in sport while furthering their education (i.e., dual-career athletes), yet there are inherent differences in the experiences of student-athletes in the United States compared to other countries. Roberts et al. (1) highlight the importance of a formalized dual-career structure when developing and training student-athletes: “the NCAA model demonstrates high interconnectivity between academic, athletic, and student support functions” (p. 649). What they found is New Zealand dual-career athletes would benefit from a similar structure, where the sport governing body could provide this support for their athletic and academic endeavors. The purpose of this research topic was to examine the multifaceted aspects of the student-athlete and dual career athlete experience. The articles within this collection demonstrate the need for understanding how academic demands interact with sport participation in a variety of ways. Three specific contexts this collection identifies as important include: cross-cultural perspectives, physical and mental well-being, organizational/institutional systems, and post-career transitions.

## Cross-cultural perspectives

In Hallmann and Weustenfeld's (2) meta-analysis, they observed a Eurocentric focus of dual-career athletes. Li et al. and Kim et al. fill this research gap by demonstrating how culture shapes the athletes' experiences. Li et al.'s research comparing participation and psychological resilience across China, the United States, and Europe demonstrates how culture can shape psychological factors like motivation and mental health of these athletes. Similarly, Kim et al. examined the influence of peer social networks on team cohesion and effectiveness among collegiate athletes in Korea and Hong Kong. They found that social network density influenced effort, ability, and preparation of these collegiate athletes. They also found that social network density and nationality influenced team effectiveness.

## Physical/emotional wellbeing, burnout

Compared to other athletes, student-athletes and dual-career athletes face additional pressures and considerations outside of the athletic context. Xu et al., Golub and Steinfeldt, Keriven et al., Galbierz, et al. and Zhuang and Wang examine some of these different pressures and considerations. Xu et al. specifically examine the relationship between academic and athletic burnout by identifying psychological skills training, such as emotional regulation and sleep quality, as mechanisms that can mediate the transference of athlete burnout to academic burnout. Keriven et al. conducted an analysis comparing the differences in psychological well-being of European and American student-athletes. Golub and Steinfeldt examined the factors present in student-athletes who experience multiple injuries. They found that emotional reactions, support networks, and coping strategies can influence recovery. One final aspect of mental health examined in this special topic is the mental health of international student-athletes enrolled at United States universities. Galbriez et al. found that international student-athletes face unique mental health risk factors, and social support/relationship quality can positively influence the mental well-being of these student-athletes. Finally, Zhuang and Wang present an autoethnographic account of a Chinese student-athlete recovering from a sport-related concussion and navigating the process of role exit from competitive sport. Their analysis illustrates how institutional resources, social support networks, and access to academic opportunities collectively influence both the recovery process and the individual's transition in identity beyond sport.

## Organizational/institutional systems

Student-athletes and dual-career athletes are similar in that they both are advancing their education as they also pursue a playing career, yet the relationship between their teams and educational institutions is different. Student-athletes in the United States are recruited by universities for their athletic abilities, while dual-career athletes are playing for an unrelated club team and simultaneously pursuing further education. Pereira da Silva et al., Al-Fattal et al., and Luo et al. examine various factors related to the pursuit of higher education and elite-level sport. Pereira da Silva et al. examine the processes by which dual-career athletes are admitted to Brazilian federal universities, and therefore, access higher education to enhance their post-career opportunities. Al-Fattal et al. conducted a qualitative analysis of the different factors influencing a student-athlete's institutional choice that are assessed during recruitment. Factors such as academic offerings/reputation, social connections, and long-term career goals played an important role in attracting student-athletes to their current institution. Finally, Luo et al. examine factors influencing perceptions and participation in esports at United States universities. Five dimensions used to assess perceptions include attraction, economics, recognition, socialization, and technicity. Understanding influences on esports perceptions and participation will help universities determine ways to use esports to enhance student learning and skill development.

## Post-career transition

When the number of student-athletes is compared with the number of those who go on to play professionally, an essential aspect of the student-athlete experience is preparing for a career after their playing career ends. Piacentine et al. examine strategies for supporting student-athletes for a career after sport. Examples of factors that would facilitate a healthier transition they suggest include “guidance on exercising for general health, managing pain and prior injuries, nutrition, and social support”. Also, Correia-Harker et al. examine the relationship between intercollegiate athletics participation and leadership development. What they found is that sport participation alone does not ensure that student-athletes will develop leadership skills, yet incorporating high-impact practices in tandem with their sport participation may help facilitate leadership skills development.

## Conclusion

This collection identifies the variety of factors associated with student-athletes and dual-career athletes. The authors here extend beyond the athletic performance of these athletes by examining the developmental, cultural, psychosocial, and organizational/institutional systems associated with supporting student-athlete and dual-career athletes across their competitive careers and beyond. Also, the methodologies used to assess these relationships were just as varied. While these studies are a part of a foundation of student-athlete research, there is more to be done.
